# Human capital, social capital, psychological capital, and job performance: Based on fuzzy-set qualitative comparative analysis

**DOI:** 10.3389/fpsyg.2022.938875

**Published:** 2022-08-10

**Authors:** Qian Xu, Zhe Hou, Chao Zhang, Feng Yu, Jiangyue Guan, Xiao Liu

**Affiliations:** ^1^School of Education Science, Shanxi Normal University, Taiyuan, China; ^2^School of Philosophy, Wuhan University, Wuhan, China

**Keywords:** human capital, social capital, psychological capital, job performance, fuzzy-set qualitative comparative analysis

## Abstract

The present study investigated the configuration effect of human capital, social capital, and psychological capital on job performance. The human capital questionnaire, social capital scale, psychological capital scale, and job performance scale were used to survey 458 employees. Results revealed that four antecedent configurations could achieve high task performance, and three antecedent configurations can achieve high contextual performance. The high job performance driving path was characterized by “all roads lead to Rome.” Human capital, social capital, and psychological capital affected job performance in the form of configuration, which reflected the asymmetric causal relationship.

## Introduction

Human resource management aims to boost productivity and increase profits. As a result, boosting performance has become a top priority. In the era of the boundaryless career, individual career development is variable, non-linear, and unpredictable, causing people to be uncertain about themselves, their identities, and their surroundings (Pryor and Bright, 2018). Intelligent career theory proposes “knowing-how” career capital, “knowing-whom” career capital, and “knowing-why” career capital to confront the uncertain career development environment (Beigi et al., 2018), which represent human capital, social capital, and psychological capital, respectively (Dickmann et al., 2018). Human capital refers to the knowledge, skills, and experience formed by investment in education and training (Goldin, 2016). Social capital is the interpersonal network formed by relationship investment (Ehsan et al., 2019). Psychological capital is an individual positive psychological ability that can be measured, developed, and can improve job performance (Nolzen, 2018).

Few studies examined the effect of three capitals on a variety of workplace outcomes simultaneously, including job performance (Huang et al., 2020), turnover intention (Li et al., 2021), and organizational commitment (Tian and Zuo, 2013). They all relied on regression analysis and assumed that the three capitals were independent of one another and that their impact on job performance was symmetrical.

However, the three capitals have a complicated connection, and the effects of the three capitals on job performance are asymmetric. We discovered that the following essential issues had not been addressed in existing research: (1) What is the interdependence of human capital, social capital, and psychological capital on job performance? (2) Does the Impact of three capitals on job performance have any asymmetric causality? The answers to the preceding questions are scientifically significant. In theory, this study examined the configuration effect and asymmetric causality of human capital, social capital, and psychological capital on job performance. It helped discover new factors affecting job performance, challenging previous studies’ symmetrical regression relationship findings and enriching the relationship among three capitals in intelligent career theory. In practice, it assists businesses in believing that people with low career capital can still achieve excellent job performance, as well as provides evidence for the company on how to attach importance to the three capitals.

Therefore, by challenging the research using traditional regression analysis, the innovation of this study is to find the configuration effect of three capitals on job performance and to explore the causal asymmetry among variables.

## Theoretical background

### Job performance

Job performance includes task performance and contextual performance (Tong, 2018). Task performance is affected by knowledge, experience, and job proficiency, and it emphasizes the completion of the specified task of the job description, which is part of the formal reward system (Tong, 2018). Contextual performance is mainly affected by motivation and personality factors. It emphasizes that employees should have a high degree of enthusiasm, the ability to implement the organizational system strictly, and the initiative to complete work beyond their posts and help others realize their work (Singh, 2019). However, contextual performance is usually not part of the formal reward system. Contextual performance emphasizes that employees do not directly participate in production or service activities but constitute psychological and social backgrounds in the organization (Budhiraja, 2021). Contextual performance promotes task performance and improves the effectiveness of the whole organization in achieving goals (Jannesari et al., 2021).

### Intelligent career theory

The intelligent career theory was first proposed to fight against boundaryless careers (Beigi et al., 2018). According to Beigi et al. (2018), the three core competencies of intelligent enterprises (i.e., the company’s “knowing-how,” social network, and organizational culture) require employees to develop “knowing-how” career capital, “knowing-whom” career capital, and “knowing-why” career capital.

“Knowing-how” refers to employees knowledge, skills, and abilities (Beigi et al., 2018), answers the question of “how do you work,” and reflects the situation of personal human capital (Parker et al., 2009). “Knowing-whom” refers to internal and external interpersonal contacts that aid the company’s social networking efforts (Beigi et al., 2018). “Knowing-whom” is a response to the question of “whom do you work with,” reflecting the individual’s social capital (Parker et al., 2009). “Knowing-why” answers the question of “why do you work,” taking into account personal motivation, identity, personality, interests, and values (Parker et al., 2009; Beigi et al., 2018), and reflects the state of positive psychological capital within individuals (Parker et al., 2009; Beigi et al., 2018).

According to intelligent career theory, employees’ human capital, social capital, and psychological capital are the three core vocational abilities for employees to be competent for their occupations and achieve core competitive advantages for their careers (Luthans et al., 2015). As individual competitive advantages, human capital, social capital, and psychological capital can achieve high job performance (Luthans et al., 2015).

### Human capital and job performance

Human capital is an economic concept that emphasizes that people, as a type of capital, have a more significant potential for appreciation than physical capital (e.g., money) (Goldin, 2016). Human capital includes explicit and implicit human capital (Luthans et al., 2015). Explicit human capital refers to the external components that make up the value of human capital and can be measured using standard methods like education and service length (Luthans et al., 2015). Implicit human capital refers to employees’ knowledge, experience, creativity, and value system (Luthans et al., 2015). Implicit human capital is more original and fundamental than explicit human capital, and it is the wellspring of innovation performance and the cornerstone of all explicit knowledge (Luthans et al., 2015).

In a meta-analysis, Ng and Feldman (2009) discovered that education level could predict creativity and task performance. Ke et al. (2010) found that human capital composed of theoretical knowledge, work experience, and professional skill level had good predictive validity for task performance and contextual performance. Furthermore, Imran and Atiya (2020) found that a high-performance work system enhanced job performance through the mediation of human capital.

### Social capital and job performance

Social capital reflects a reciprocal benefit relationship. With the help of this social relationship, individuals can utilize other external resources such as information and knowledge to make up for the lack of resources (Tulin et al., 2018). Two fundamental indexes can quantify social capital: one that reflects individual social network structure, such as network size, network quality, and relationship quality (Lin, 2017). The other measures how individuals utilize social relationships, such as mobilized social capital (Lin, 2017).

Rich social capital is essential to work since employees’ job is directly or indirectly tied to others. Employees must frequently communicate and collaborate effectively with leaders, coworkers, and subordinates to complete work. Individuals with rich social capital are more likely to receive outside assistance. According to the theory of social exchange and social norms, individuals with rich social capital will be more helpful and have more work enthusiasm and dedication to repay others’ support and keep social networks alive (Wang et al., 2019). As a result, rich social capital helps to improve task performance and contextual performance. Ke et al. (2010) also found that social capital can effectively improve employee task and contextual performance.

### Psychological capital and job performance

Psychological capital is a measurable, developable, and motivating individual positive mental capacity that contributes to job performance (Luthans et al., 2015). Psychological capital is generally a four-dimensional model, including self-confidence, optimism, resiliency, and hope (Luthans et al., 2015). Under the Chinese organizational situation, Ke et al. (2009) compiled a scale of Chinese psychological capital, broadening the meaning of psychological capital and dividing psychological capital into task-oriented psychological capital and *guanxi*-oriented psychological capital.

Through meta-analysis, Avey et al. (2011) found that psychological capital can effectively improve employees’ job performance. Through longitudinal studies, Peterson et al. (2011) discovered that psychological capital can continue to impact job performance over time positively. Furthermore, numerous studies have discovered that psychological capital can boost academic performance (Martínez et al., 2019), firm performance (Grözinger et al., 2022), creative performance (Ozturk and Karatepe, 2019), and nursing performance (Nasurdin et al., 2018; Ozturk and Karatepe, 2019).

### Configuration effect of human, social, and psychological capital on job performance

Individual career competitiveness, human capital, social capital, and psychological capital are conducive to improving job performance, according to intelligent career theory (Parker et al., 2009). Human capital includes explicit human capital (e.g., education level) and implicit human capital (e.g., internal knowledge and skills) (Luthans et al., 2015). Social capital includes interpersonal and group relationships, potential groups, community resources, and social structures (Luthans et al., 2015). Psychological capital emphasizes positive psychological qualities, including self-confidence, optimism, resiliency, and hope (Luthans et al., 2015).

Previous studies have shown human capital (Huang et al., 2020), social capital (Clausen et al., 2019; Yen et al., 2020), and psychological capital (Ali et al., 2022; Qasim et al., 2021) can positively improve job performance, respectively. Ke et al. (2010) and Huang et al. (2020) used regression analysis to examine the simultaneous effect of three capitals on job performance. Moreover, Ke et al. (2010) found that psychological capital had the most significant impact on employees’ task performance and contextual performance, followed by social and human capital. Huang et al. (2020) revealed that psychological capital significantly impacted hotel employees’ self-rated job performance, followed by explicit human and social capital. However, only explicit human capital could increase other-rated job performance significantly.

Multiple regression analysis was previously used in research, which presupposes that independent variables are independent of one another and that there is causal symmetry between variables (Rihoux and Ragin, 2008). Based on these hypotheses, human capital, social capital, and psychological capital affect job performance independently. Furthermore, the effects of three capitals on job performance do not have the phenomenon of “the same for different fruit” and “all roads lead to Rome.” Nevertheless, in essence, the relationship between human capital, social capital, and psychological capital does not accord with the hypotheses of multiple regression.

Firstly, there is a conceptual overlap among the three capitals. For example, moral meanings (e.g., norms, values, and integrity) are included in social capital (Luthans et al., 2015), while morality is also the spiritual level of human capital (Wang, 2018). Furthermore, trust in social capital (Luthans et al., 2015) and modesty in psychological capital (Ke et al., 2009) are linked to good interpersonal relationships.

Secondly, the three capitals are interdependent. There is a pairwise correlation and a complex synergistic mechanism among the three capitals (Beigi et al., 2018). Psychological capital can promote social capital formation through the contagion effect, while social capital can strengthen employees’ psychological capital by solidifying employees’ psychological contracts (Zhang and Wu, 2009). Furthermore, psychological capital influences an individual’s subjective career success via the mediation of human and social capital (Zhou et al., 2015). More human capital facilitates forming more interpersonal relationships (Parker and Arthur, 2015). Task-oriented psychological capital and *guanxi*-oriented psychological capital interact with organizational commitment (Ke and Sun, 2014), work engagement, and subjective well-being (Ke et al., 2015). *Guanxi*-oriented psychological capital contributes to the buildup of social capital by removing barriers to long-term interpersonal relationships, open communication, knowledge exchange, and continual feedback (Luthans et al., 2015). Moral capital is the spiritual level of human capital. The improvements in moral capital may foster an enterprising spirit (psychological capital) and harmonious cooperation among people (social capital and *guanx*i-oriented psychological capital) (Wang, 2018).

Furthermore, multiple regression analysis assumes causal symmetry among variables, not accounting for the fact that three capitals affect job performance with multiple conjunctural causation (Rihoux and Ragin, 2008). For example, Huang et al. (2020) found that high explicit human capital (e.g., education level and length of service) led to high job performance, and low explicit human capital led to low job performance. However, in the actual workplace, low explicit human capital can also result in high job performance. Thus, the causal symmetry assumption of multiple regression analysis ignores the multiple conjunctural causation, causing the deviation between results and reality and eventually misleading the management practice.

To explore the causal complexity among variables, fuzzy-set qualitative comparative analysis (fsQCA) ignores some core assumptions of traditional regression, such as independence between prediction variables, homogeneity of analysis units, constancy, and causal symmetry. FsQCA emphasizes the multiple conjunctural causation, which is non-linear and non-constant. It highlights the phenomenon of “all roads lead to Rome,” the complex configuration of antecedent conditions, and the diverse characteristics. FsQCA can detect the set relationship between configuration and outcome variable, allowing it to overcome the above defects associated with multiple regressions (Rihoux and Ragin, 2008). If there are numerous contradicting configurations, the theory or hypothesis should be dismissed (Rihoux and Ragin, 2008).

Therefore, this study used fsQCA to investigate the complicated set relationship and asymmetric causation among three capitals and job performance from a configuration perspective, which was different from previous research. In theory, it is possible to overcome the limitations of previous research and enrich the content of intelligent career theory by discovering the causal complexity. In practice, it is helpful for employers and employees to adopt more targeted investment strategies for three capitals and to obtain the maximum performance improvement through the lowest resource investment cost.

## Materials and methods

### Participants and procedure

The participants were selected by convenient sampling and came from 26 enterprises in nine provinces of China, involving 13 industries such as aquaculture, new energy, and food processing. Distribute paper self-report questionnaire on site.

This study set some anti-counterfeiting items, such as “Please directly select “fully agree.”” If the item was selected incorrectly, it would be regarded as invalid data. Moreover, questionnaires with regular answers and too many missing values were also regarded as invalid data. Among 648 distributed questionnaires, 458 valid questionnaires were recovered, with an effective rate of 70.68%.

### Human capital questionnaire

The *Human Capital Questionnaire* is a three-item scale created by Ke et al. (2010). The responses to statements are rated on a 6-point Likert scale. Cronbach’s α for the scale was 0.89.

### Social capital scale

The *Social Capital Scale* is a 23-item scale created by Wang and Long (2009). The scale has two subscales: accessed social capital and mobilizing social capital, whereas the former includes network size, network diversity, and social source. Mobilized social capital includes relatives’ career sponsorship, friends’ career sponsorship, and acquaintances’ career sponsorship (Wang and Long, 2009). The responses to statements are rated on a 6-point Likert scale. Cronbach’s αs for the total scale and subscales were 0.95, 0.92, and 0.94, respectively. Using confirmatory factor analysis (CFA) to test construct validity, χ^2^/*df* = 3.53, TLI = 0.92, CFI = 0.93, RMSEA = 0.07, RMR = 0.051, GFI = 0.87, PNFI = 0.72.

### Chinese psychological capital scale

The *Chinese Psychological Capital Scale* is a 40-item scale created by Ke et al. (2009). The scale has two subscales: task-oriented psychological capital and *guanxi*-oriented psychological capital. Task-oriented psychological capital includes self-confidence and courage, optimism and hope, the spirit of enterprise and diligence, resiliency and perseverance. *Guanxi*-oriented psychological capital includes toleration and forgiveness, respect and courtesy, modesty and prudence, thankfulness and dedication (Ke et al., 2009). The responses to statements are rated on a 6-point Likert scale. Cronbach’s αs for the total scale and subscales were 0.96, 0.94, and 0.93, respectively. Using CFA to test construct validity, χ^2^/ *df* = 2.310, TLI = 0.91, CFI = 0.92, RMSEA = 0.05, RMR = 0.05, GFI = 0.85, PNFI = 0.77.

### Job performance scale

The *Job Performance Scale* is a 10-item scale created by Van Scotter (2000). The scale has two subscales: task performance and contextual performance. The responses to statements are rated on a 5-point Likert scale. Cronbach’s αs for the total scale and subscales were 0.91, 0.87, and 0.88, respectively. Using CFA to test construct validity, χ^2^/*df* = 5.60, TLI = 0.92, CFI = 0.94, RMSEA = 0.10, RMR = 0.04, GFI = 0.91, PNFI = 0.70.

### Statistical analysis

Fuzzy-set qualitative comparative analysis is based on Boolean logic analysis and can only deal with fuzzy-set membership scores between 0 and 1. The fuzzy membership score “1” represents “completely belonging to a set,” “0” represents “completely not belonging to a set,” and “0.5” is the maximum fuzzy point when evaluating whether a case belongs to or does not belong to a set.

Since the Likert scale data does not meet the conditions for Boolean logistic analysis, the score was calibrated by fsQCA 3.1, and the raw data were converted into collective data between 0 and 1. The human capital scale, social capital scale, and Chinese psychological capital scale were scored by Likert 6-point, so “1” was defined as complete non-membership, “6” as complete membership, and “3.5” as the maximum fuzzy point. The job performance scale was scored by 5-point Likert, where “1” was defined as complete non-membership, “5” as complete membership, and “3” as the maximum fuzzy point. Through the setting of three thresholds, the scale score was converted into a fuzzy membership degree between 0 and 1. Then, calculate the fuzzy intersection of task performance * contextual performance through the ‘fuzzy and’ statement (“*” represents logic “and”). The fsQCA3.1 software was used to analyze the necessity of antecedent conditions, construct the truth table, and perform a standard analysis of antecedent configuration.

## Results

### Descriptive and correlations analysis

The results of descriptive statistics and correlation analysis are shown in Table 1. A significant positive correlation was found between the variables, indicating an interaction between them. On this basis, the configuration effects of the three capitals on job performance were investigated.

**TABLE 1:** Descriptive statistics and correlation analysis of variables.

Variable	*x*	*s*	1	2	3	4	5	6
Human capital	4.12	1.07						
Accessed social capital	3.90	0.93	0.45**					
Mobilized social capital	3.91	0.97	0.36**	0.80**				
Task-oriented psychological capital	4.75	0.70	0.38**	0.48**	0.44**			
*Guanxi*-oriented psychological capital	4.79	0.60	0.32**	0.46**	0.47**	0.76**		
Task performance	4.22	0.59	0.38**	0.25**	0.19**	0.50**	0.43**	
Contextual performance	4.12	0.65	0.43**	0.43**	0.41**	0.56**	0.53**	0.68**

**p < 0.01.

### Necessity analysis

The necessity of each antecedent condition to the outcome variable was analyzed (Table 2). When the job performance was high, the consistencies of high task-oriented and *guanxi*-oriented psychological capital were always in the range of 0.92-0.98 (>0.9). It showed that high psychological capital is necessary to achieve high job performance. The consistency of other antecedent conditions was <0.9, which did not constitute a necessary condition.

**TABLE 2:** Necessity analysis of antecedent conditions for job performance.

Antecedent condition	High task performance		High contextual performance		High task performance * High contextual performance	
			
	Consistency	Coverage	Consistency	Coverage	Consistency	Coverage
High human capital	0.75	0.98	0.77	0.97	0.79	0.96
Non-high human capital	0.42	0.96	0.42	0.94	0.43	0.93
High accessed social capital	0.70	0.98	0.72	0.98	0.74	0.97
Non-high accessed social capital	0.47	0.97	0.47	0.95	0.49	0.94
High mobilized social capital	0.70	0.97	0.72	0.97	0.74	0.96
Non-high mobilized social capital	0.47	0.97	0.47	0.94	0.48	0.94
**High task-oriented psychological capital**	**0.97**	**0.93**	**0.97**	**0.91**	**0.98**	**0.89**
Non-high task-oriented psychological capital	0.16	0.99	0.17	0.99	0.17	0.98
**High *guanxi*-oriented psychological capital**	**0.92**	**0.96**	**0.94**	**0.94**	**0.95**	*0.92*
Non-high *guanxi*-oriented psychological capital	0.24	0.99	0.24	0.98	0.25	0.98

The bold values represent the relevant consistency and coverage results of the consistency conditions (i.e., task-oriented and guanxi-oriented psychological capital).

### Configurations for job performance

Fuzzy-set qualitative comparative analysis 3.1 incorporated various antecedent conditions into standard analysis to analyze the configuration solutions affecting job performance. Since the sample size was 458, the frequency threshold was set to 3 (Fiss, 2011). Since the consistency of all truth table rows was >0.8, and the PRI consistency (Proportional Reduction in Inconsistency) was >0.75, each configuration had a strong subset relationship with the outcome variable, so configuration could not be further filtered. Thus, the natural breaks of consistency and PRI consistency were considered. The truth table row under the slightest natural break was coded as 0 to retain more truth table rows. Considering the standard analysis results, as the conclusion obtained by the intermediate solution was more enlightening and universal than the complex and parsimonious solutions, the results of the intermediate solution were listed.

The results in Table 3 showed that the antecedent configuration for high task performance included four combinations, with an overall consistency of 0.98 and an overall coverage rate of 0.81, which indicated that the overall configuration provided a convincing explanation for the results. The four configurations were as follows: configuration A (non-high accessed social capital * high task-oriented psychological capital * high *guanxi-*oriented psychological capital), configuration B (non-high mobilized social capital * high task-oriented psychological capital * high *guanxi*-oriented psychological capital), configuration C (high human capital * high task-oriented psychological capital * high *guanxi*-oriented psychological capital), and configuration D (high human capital * non-high accessed social capital * non-high mobilized social capital * high task-oriented psychological capital). The consistency of the four configurations was 0.99, which indicated that with this configuration, the case had a 99% probability of achieving high task performance. Configuration C’s unique coverage was 0.30, indicating that 30% of the cases could only be explained by configuration C. Configuration C had the broadest raw and unique coverage, indicating that configuration C explained the largest number of cases and was the most critical path to achieving high task performance. On the contrary, configuration D had the least raw coverage, reflecting that it explained the minor cases and was a secondary factor in achieving high task performance.

**TABLE 3:** Configurations for achieving high task performance.

Number	Configuration	Raw coverage	Unique coverage	Consistency	Overall solution coverage	Overall solution consistency
A	Non-high accessed social capital *High task-oriented psychological capital *High *guanxi*-oriented psychological capital	0.46	0.02	0.99	0.81	0.98
B	Non-high mobilized social capital *High task-oriented psychological capital *High *guanxi*-oriented psychological capital	0.46	0.01	0.99		
C	High human capital *High task-oriented psychological capital *High *guanxi*-oriented psychological capital	0.73	0.30	0.99		
D	High human capital *Non-high accessed social capital *Non-high mobilized social capital *High task-oriented psychological capital	0.35	0.01	0.99		

The antecedent conditions that do not appear in the configuration can exist or not exist simultaneously. The symbol “*” represents logic “and”.

The results in Table 4 showed that the antecedent configuration of contextual performance included three combinations; the overall consistency was 0.98, and the overall solution coverage was 0.81, which indicated that the overall configuration provided a convincing explanation of the results. The three configurations were: configuration E (high accessed social capital * high task-oriented psychological capital * high *guanxi*-oriented psychological capital), configuration F (high mobilized social capital * high task-oriented psychological capital * high *guanxi*-oriented psychological capital), and configuration G (high human capital * non-high accessed social capital * non-high mobilized social capital * high task-oriented psychological capital). The consistencies of the three configurations were 0.99, 0.98, and 0.99, respectively, indicating that when the configuration existed, the cases had 99, 98, and 99% possibilities to achieve high contextual performance, respectively. Configurations E and F’s raw coverage was equal, and configuration F’s unique coverage was slightly larger than that of configuration E. Thus, configuration F had a slightly more significant effect on high task performance than configuration E. Configuration G had the least raw coverage, indicating that configuration G explained fewer cases and was a secondary factor achieving high contextual performance.

**TABLE 4:** Configurations for the achievement of high contextual performance.

Number	Configuration	Raw coverage	Unique coverage	Consistency	Overall solution coverage	Overall solution consistency
E	High accessed social capital *High task-oriented psychological capital *High *guanxi*-oriented psychological capital	0.72	0.04	0.99	0.81	0.98
F	High mobilized social capital *High task-oriented psychological capital *High *guanxi*-oriented psychological capital	0.72	0.05	0.98		
G	High human capital *Non-high accessed social capital *Non-high mobilized social capital *High task-oriented psychological capital	0.36	0.04	0.99		

The antecedent conditions that do not appear in the configuration can exist or not exist simultaneously. The symbol “*” represents logic “and”.

The results in Table 5 showed that the antecedent configuration of job performance (i.e., task performance * contextual performance) included three combinations. The three configurations were: configuration H (high accessed social capital * high task-oriented psychological capital * high guanxi-oriented psychological capital), configuration I (high mobilized social capital * high task-oriented psychological capital * high guanxi-oriented psychological capital), and configuration J (high human capital * non-high accessed social capital * non-high mobilized social capital * high task-oriented psychological capital). The antecedent configurations of job performance (i.e., task performance * contextual performance) were the same as that of contextual performance, and the coverage and consistency of each configuration and the total were almost the same.

**TABLE 5:** Configurations for the achievement of high contextual performance * high contextual performance.

Number	Configuration	Raw coverage	Unique coverage	Consistency	Overall solution coverage	Overall solution consistency
H	High accessed social capital *High task-oriented psychological capital *High *guanxi*-oriented psychological capital	0.73	0.04	0.97	0.83	0.96
I	High mobilized social capital *High task-oriented psychological capital *High *guanxi*-oriented psychological capital	0.73	0.05	0.97		
J	High human capital *Non-high accessed social capital *Non-high mobilized social capital *High task-oriented psychological capital	0.37	0.04	0.99		

The antecedent conditions that do not appear in the configuration can exist or not exist simultaneously. The symbol “*” represents logic “and”.

**FIGURE 1:**
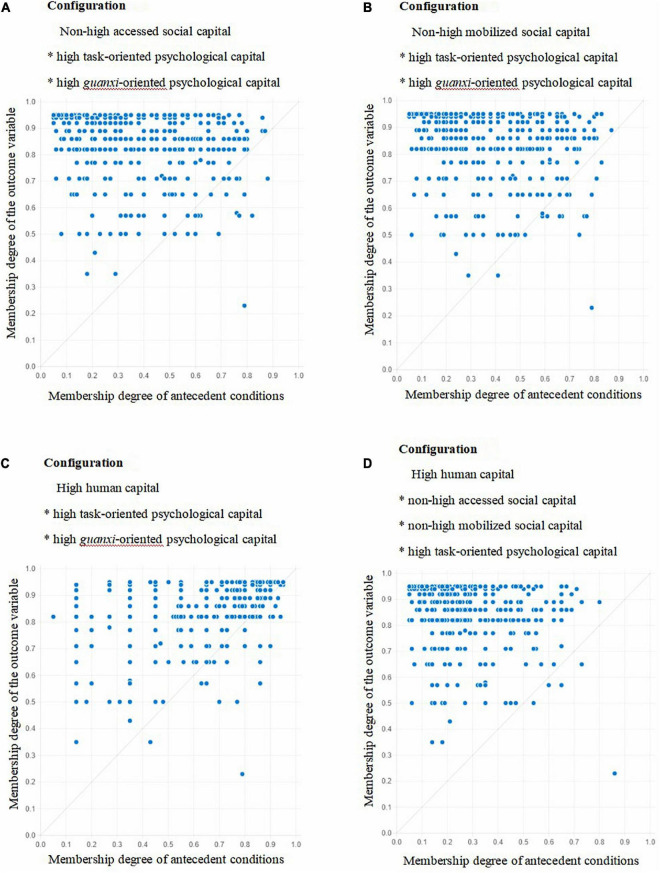
Fuzzy subset relation diagrams for Configurations **A–D**, and task performance.

**FIGURE 2:**
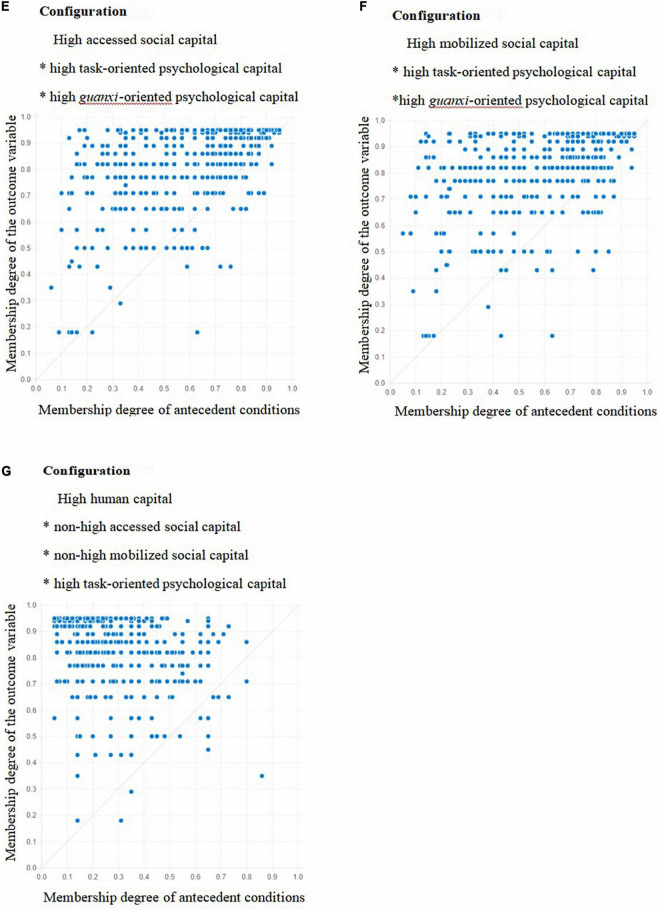
Fuzzy subset relation diagrams for Configurations **E–G**, and contextual performance.

**FIGURE 3:**
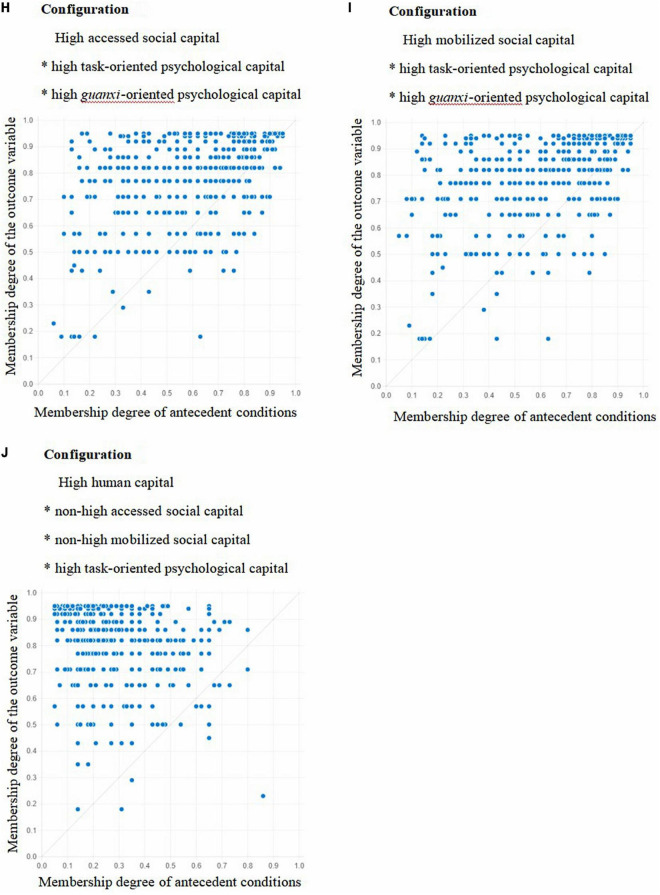
Fuzzy subset relation diagrams for Configurations **H–J**, and task performance * contextual performance.

By fitting the sample data, the X-Y plots of 10 configurations where high task performance, high contextual performance, and high task performance * high contextual performance were achieved were obtained, as shown in Figures 1–3. Figures 1–3 showed that when the membership degree of condition configuration was high, it could produce high job performance; When the membership degree of condition configuration was low, it could also produce high job performance. Therefore, there was a causal asymmetry between condition configuration and high job performance, which could not be obtained by traditional regression analysis.

## Discussion

### Configurations to achieve high job performance

High task-oriented psychological capital and high *guanxi*-oriented psychological capital are the necessary conditions affecting high job performance, thus suggesting that high psychological capital is the most critical factor for high job performance. It is consistent with Ke et al. (2010) and Xu et al. (2021), who argued that psychological capital is the most critical factor influencing job performance and well-being. High task-oriented psychological capital was found in all antecedent configurations of high job performance, reflecting the task-oriented psychological capital as a necessary condition to achieve high job performance. Although high *guanxi*-oriented psychological capital was necessary for high job performance, its consistency did not reach 1. So, even if *guanxi*-oriented psychological capital is very important, it can not necessarily exist in configurations D and G. Thus, high task-oriented psychological capital has a greater effect on high job performance than high *guanxi*-oriented psychological capital. Compared with high *guanxi*-oriented psychological capital, the achievement of high job performance was found to depend more on high task-oriented psychological capital.

Among the four antecedent configurations of high task performance, the raw coverage of configurations A and B was equal, and configuration A’s unique coverage was slightly more significant than configuration B’s. It indicated that compared with mobilized social capital, psychological capital could better compensate for lacking accessed social capital on high task performance. It is supported by correlation analysis, which found that accessed social capital and task performance had a higher correlation coefficient than mobilized social capital and task performance. Configuration A, B, and C showed that with high task-oriented psychological capital and high *guanxi*-oriented psychological capital, high human capital made the unique coverage of configuration C much more significant than other configurations. Even if high mobilized social capital was found in configuration A and high accessed social capital was found in configuration B, the coverage of configuration A and B was lower than configuration C, indicating that high human capital has a more significant role in high task performance than high social capital. Configuration C and D showed that the effect of high human capital on high task performance could only be exerted when task-oriented psychological capital is high. So, the effect of high human capital on high task performance depends on high task-oriented psychological capital, not high *guanxi*-oriented psychological capital. At the same time, the four configurations showed that the absence of social capital could also achieve high task performance, reflecting causal asymmetry.

Among the antecedent configurations of high contextual performance, configurations E and F showed that even if human capital is lacking, as long as social capital and psychological capital are high, and it could lead to high contextual performance. Configuration E and F’s raw coverage was equal, and configuration F’s unique coverage was slightly more significant than configuration E’s. Thus, the effect of high mobilized social capital on high contextual performance was slightly greater than that of high accessed social capital. However, the results of correlation analysis showed that the correlation coefficient between social capital and contextual performance was more significant than that between accessed social capital and contextual performance. This difference was related to the fact that correlation analysis did not consider asymmetric causality. Configuration G showed that when both kinds of social capital were lacking, high contextual performance could be achieved as long as human capital and task-oriented psychological capital were high. Configurations E, F, and G showed that high contextual performance could also be achieved with the lack of social capital. Nonetheless, the number of cases lacking the two types of social capital was small. Moreover, high task-oriented psychological capital is a more important factor affecting high contextual performance. The impact of high *guanxi*-oriented psychological capital on high contextual performance depends on social capital. In three configurations, when task-oriented psychological capital was high, even if *guanxi*-oriented psychological capital was high, human capital made the coverage of configuration G lower than configurations E and F of high social capital. It indicated that the effect of human capital on contextual performance was smaller than social capital. It is consistent with the findings of Ke et al. (2010), suggesting that social capital had a more significant effect on contextual performance than human capital.

By taking the fuzzy intersection of task performance * contextual performance, configurations E, F, and G were still the antecedent configurations when the two kinds of job performance were both achievings. Therefore, configurations E, F, and G were not only the configuration to achieve high contextual performance but also the configuration to achieve two kinds of job performance simultaneously.

The two kinds of social capital could be lacking in the antecedent configurations of high task performance but exist in several antecedent configurations of high contextual performance. Thus, compared with high task performance, the achievement of high contextual performance was more dependent on social capital. It is consistent with the correlation coefficient, i.e., although the correlation coefficients between task performance and two types of social capital reached a significant level, they were lower than the correlation coefficients between contextual performance and two types of social capital.

### Management implications

Managers should recognize that even if employees have low career capital, they can still achieve high job performance. It requires managers to invest in employees’ three capitals to improve job performance most effectively.

Employers and employees should focus on investing in psychological capital, especially task-oriented psychological capital, and cultivate the positive qualities of self-confidence and courage, optimism and hope, the spirit of enterprise and diligence, resiliency, and perseverance. When improving task performance, it is necessary to focus on psychological capital, human capital, and social capital, respectively; when improving contextual performance, it is necessary to pay attention to psychological capital, social capital, and human capital, respectively. When the two dimensions of job performance are not distinguished, human capital and social capital, as well as accessed social capital and mobilized social capital, should be considered equally important.

## Conclusion

(1) Human capital, social capital, and psychological capital affect job performance in the form of configuration, and there is causal asymmetry. High psychological capital is the most critical factor in achieving high job performance. The antecedent configurations of high contextual performance are the same as when both performances are high. (2) The effect of high human capital on high task performance is greater than that of high social capital and depends on high task-oriented psychological capital. Compared with mobilized social capital, high psychological capital can make up more for the lack of accessed social capital on high task performance. (3) The Impact of high social capital on high contextual performance is more significant than that of high human capital. The impact of high mobilization social capital on high contextual performance is slightly more significant than that of high exposure social capital. In the case of high psychological capital and lack of human capital, high contextual performance can be produced in accessed social capital or high mobilization social capital. High human capital and high task-oriented psychological capital can achieve high contextual performance. Moreover, the influence of high *guanxi*-oriented psychological capital on high contextual performance depends on high social capital.

## Data availability statement

The original contributions presented in this study are included in the article/Supplementary material, further inquiries can be directed to the corresponding author.

## Ethics statement

The studies involving human participants were reviewed and approved by Department of Psychology and Institute of Applied Psychology, Shanxi Normal University. The patients/participants provided their written informed consent to participate in this study. Written informed consent was obtained from the individual(s) for the publication of any potentially identifiable images or data included in this article.

## Author contributions

QX and ZH contributed to conceptualization, data collection and analysis, and writing. The first draft of the manuscript was written by QX and CZ. All authors commented on previous versions of the manuscript and read and approved the final manuscript.

## Conflict of interest

The authors declare that the research was conducted in the absence of any commercial or financial relationships that could be construed as a potential conflict of interest.

## Publisher’s note

All claims expressed in this article are solely those of the authors and do not necessarily represent those of their affiliated organizations, or those of the publisher, the editors and the reviewers. Any product that may be evaluated in this article, or claim that may be made by its manufacturer, is not guaranteed or endorsed by the publisher.
